# Altered IL-7 signaling in CD4^+^ T cells from patients with visceral leishmaniasis

**DOI:** 10.1371/journal.pntd.0011960

**Published:** 2024-02-26

**Authors:** Shashi Kumar, Shashi Bhushan Chauhan, Shreya Upadhyay, Siddharth Sankar Singh, Vimal Verma, Rajiv Kumar, Christian Engwerda, Susanne Nylén, Shyam Sundar

**Affiliations:** 1 Department of Medicine, Institute of Medical Sciences, Banaras Hindu University, Varanasi Uttar Pradesh India; 2 School of Medicine & Health Sciences, The George Washington University, Washington, Washington, United States of America; 3 University of Massachusetts Chan Medical School, Shrewsbury, Massachusetts, United States of America; 4 Centre of Experimental Medicine and Surgery, Banaras Hindu University, Varanasi, India; 5 QIMR Berghofer Medical Research Institute, Brisbane, Australia; 6 Department of Microbiology, Tumor and Cell Biology, Karolinska Institutet, Stockholm, Sweden; Ohio State University, UNITED STATES

## Abstract

**Background:**

CD4^+^ T cells play a central role in control of *L*. *donovani* infection, through IFN-γ production required for activation of macrophages and killing of intracellular parasites. Impaired control of parasites can in part be explained by hampered CD4^+^ T cells effector functions in visceral leishmaniasis (VL) patients. In a recent studies that defined transcriptional signatures for CD4^+^ T cells from active VL patients, we found that expression of the IL-7 receptor alpha chain (IL-7Rα; CD127) was downregulated, compared to CD4^+^ T cells from endemic controls (ECs). Since IL-7 signaling is critical for the survival and homeostatic maintenance of CD4^+^ T cells, we investigated this signaling pathway in VL patients, relative to ECs.

**Methods:**

CD4^+^ T cells were enriched from peripheral blood collected from VL patients and EC subjects and expression of *IL7* and *IL7RA* mRNA was measured by real time qPCR. IL-7 signaling potential and surface expression of CD127 and CD132 on CD4^+^ T cell was analyzed by multicolor flow cytometry. Plasma levels of soluble IL-7 and sIL-7Rα were measured by ELISA.

**Result:**

Transcriptional profiling data sets generated previously from our group showed lower *IL7RA* mRNA expression in VL CD4^+^ T cells as compared to EC. A significant reduction was, however not seen when assessing *IL7RA* mRNA by RT-qPCR. Yet, the levels of soluble IL-7Rα (sIL-7Rα) were reduced in plasma of VL patients compared to ECs. Furthermore, the levels of soluble IL-7 were higher in plasma from VL patients compared to ECs. Interestingly, expression of the IL-7Rα protein was higher on VL patient CD4^+^ T cells as compared to EC, with activated CD38^+^ CD4^+^ T cells showing higher surface expression of IL-7Rα compared to CD38^-^ CD4^+^ T cells in VL patients. CD4^+^ T cells from VL patients had higher signaling potential baseline and after stimulation with recombinant human IL-7 (rhIL-7) compared to EC, as measured by phosphorylation of STAT5 (pSTAT5). Interestingly, it was the CD38 negative cells that had the highest level of pSTAT5 in VL patient CD4^+^ T cells after IL-7 stimulation. Thus, despite unaltered or potentially lowered *IL7RA* mRNA expression by CD4^+^ T cells from VL patients, the surface expression of the IL-7Rα was higher compared to EC and increased pSTAT5 was seen following exposure to rhIL-7. Accordingly, IL-7 signaling appears to be functional and even enhanced in VL CD4^+^ T cells and cannot explain the impaired effector function of VL CD4^+^ T cells. The enhanced plasma IL-7 may serve as part of homeostatic feedback mechanism regulating *IL7RA* expression in CD4^+^ T cells.

## Author summary

In visceral leishmaniasis (VL), antigen specific CD4^+^ T cell responses are muted hindering the control of the *Leishmania donovani* infection. IL-7 signaling is crucial for CD4^+^ T cell survival and function, and gene expression analysis indicated that the IL-7 pathway could be altered in VL. Thus, we investigate if impaired IL-7 signaling could explain the loss of antigen specific T cell response in VL. Although we didn’t observe significant reduction of *IL7RA* mRNA by RT-qPCR, yet, the levels of soluble IL-7Rα (sIL-7Rα) were reduced in plasma of VL patients compared to ECs. Furthermore, the levels of soluble IL-7 were higher in plasma from VL patients compared to ECs and their CD4^+^ T cells exhibited heightened IL-7 receptor protein expression. Surprisingly, VL patient CD4^+^ T cells showed increased IL-7 signaling potential, as evidenced by higher phosphorylation of STAT5 upon IL-7 stimulation. While altered, the findings presented here do not attributed the impaired effector function of VL CD4^+^ T cells to defective IL-7 signaling. We speculate that the elevated plasma IL-7 is part of a homeostatic feedback mechanism in response to the reduced IL7RA transcription in CD4^+^ T cells.

## Introduction

Leishmaniasis are a parasitic disease caused by protozoan parasites of the *Leishmania* genus. All *Leishmania spp* are transmitted through the bite of infected female *Phlebotomine* sandflies. The disease can manifest in different ways, from life threating visceral leishmaniasis (VL) to localized cutaneous disease depending on the species of *Leishmania* involved. To date, around 20 different species of *Leishmania* have been identified. Each year, there are approximately 50 000–90 000 new cases of VL [1], with most cases coming from Brazil, Ethiopia, India, South Sudan, and Sudan. The most frequent manifestation of VL is anemia, and early symptoms may also include leucopenia [2,3]. Other clinical symptoms of VL include prolonged fever, enlarged spleen and liver, weight loss, and polyclonal hypergammaglobulinemia (IgG and IgM) [4]. CD4^+^ T-helper (Th) cells are central in orchestrating immune responses against *Leishmania* parasites. Specifically, T-bet^+^ CD4^+^ T cells (Th1 cells), play a crucial role in controlling *Leishmania* infection, by production of IFN-γ leading to activation of macrophages and killing of intracellular parasites [5]. However, CD4^+^ Th cells also play a role in regulating the balance between pro-inflammatory and anti-inflammatory responses, and regulatory cytokines such as IL-10 which are critical for controlling the immune response also inhibit macrophage functions and facilitate *Leishmania* infection [6,7].

IL-7 is produced by nonhematopoietic cells (e.g. stromal cells, bone marrow- mesenchymal stem cells, keratinocytes, neurons, epithelial cells, and hepatocytes) as well as dendritic cells, and plays a crucial role in supporting hematopoiesis [8]. T cells rely on this cytokine for their development, survival, and memory formation. Production of IL-7 is stimulated by various factors, including inflammation, tissue damage, and immune cell interactions [9]. IL-7 exerts its effects by binding to the IL-7 receptor (IL-7Rα), a heterodimer composed of a high-affinity CD127 (IL-7Rα) subunit and the common cytokine gamma-chain CD132. The latter is also used by other cytokines including IL-2, IL-4, IL-9, IL-15, and IL-21 [10]. Upon the binding of IL-7 to the IL-7Rα, a series of intracellular signaling events are triggered. The exact signaling pathway varies depending on cell type, but generally involves activation of Janus kinases (JAKs) and signal transducer and activator of transcription (STAT) proteins. The JAKs phosphorylate tyrosine residues on IL-7Rα, creating docking sites for STAT5 and phosphorylation of the STAT5 protein, which then as a homodimer translocate to the nucleus to modulate gene expression, thus phosphorylated STAT5 (pSTAT5) is often used to assess IL-7 signaling capacity. The activity of IL-7 is tightly regulated to maintain immune cell homeostasis. Negative regulators, such as suppressor of cytokine signaling (SOCS) proteins and protein inhibitors of activated STATs (PIAS), help to dampen IL-7 signaling and prevent excessive immune responses [11]. Increased expression of IL-7Rα on naive (TN) and memory (TM) T cells aids in the clearance of excess soluble IL-7 [12]. Once the peripheral T cell pool reaches a critical size, a balance is achieved between IL-7 consumption and production, preventing the survival of additional T cells and maintaining T cell homeostasis [12–14]. Administration of IL-7 can potentially enhance the function of immune cells and allow a larger lymphocyte pool to develop *in vivo*, and when used as an adjuvant in immunizations, IL-7 has been shown to improve long-term, antigen-specific T cell responses [15], However, dysregulation of the IL-7 pathway can contribute to the development of cancer [16].

In a previous studies, we observed a decrease in *IL7RA* expression in CD4^+^ T cells from individuals with VL compared to ECs [17,18], which suggested that IL-7 signaling could be impaired in VL patients. To gain a better understanding of the role of IL-7 in VL patients and if IL-7 played a role in VL pathogenesis and CD4^+^ T cell dysfunction, we analyzed mRNA and protein expression of IL-7 and IL-7Rα, and the ability of the IL-7 receptor to signal in PBMC and CD4^+^ T cells from VL patients and ECs.

We found a divergence between the *IL7RA* mRNA and protein expression, with an increase in IL-7Rα surface protein on VL patient CD4^+^ T cells compared to ECs. The levels of IL-7 were higher, while the levels of soluble IL-7Rα were lower in VL patient serum, compared to ECs. Moreover, activation of VL patient PBMCs showed that IL-7 signaling was functional and even enhanced in VL patient CD4^+^ T cells. In conclusion, while our data show clear differences between VL patients and ECs in regard to IL-7 and IL-7Rα levels, we cannot explain the impaired CD4^+^ T cell responses seen in VL patients by lack of IL-7 or its signaling capacity.

## Methods

### Ethics statements

All experiments were performed in accordance with the Helsinki declaration for use of human subjects in research and approval from the ethical committee of Institute of Medical Science, Banaras Hindu University-India (ethical approval No. Dean/2019/EC/1001 Dated 18/01/2019). All the participants provided written informed consent and in case of children consent was obtained from their parents or legal guardian. All subjects selected were human immunodeficiency virus negative and above 12 years of age.

### Research subjects

The following groups were included in this study: VL patients before treatment (VL, n = 128) and 30 days post treatment (VL D30) (n = 13) and endemic healthy control (ECs) subjects (n = 104) [19,20]. All donors were recruited from the Kala-Azar Medical Research Center (KAMRC), Muzaffarpur, Bihar India. The numbers of individuals indicated for each group are the total numbers included in the study, the number of donors included in each experiment is indicated in the figure legend. There was no intentional selection of the donors included in each experiment; this was based on the order of which the experiments were done and the patients available at the time. Diagnosis of VL was made based on clinical symptoms consistent with VL and detection of anti-leishmanial antibodies in serum by recombinant K39-test and/or detection of amastigote in bone marrow/splenic aspirates by microscopy [21,22]. Clinical data from the patients are summarized in Table 1.

**Table 1:** Demographic and clinical information on study participants.

Variables	EC Group	VL D0 Group	VL D30 Group
	(n = 104)	(n = 128)	(n = 13)
Age, Year			
Mean ± SD	33.6 ± 12.1	32.5 ± 13.8	24.0 ± 14.4
Median	35.0	35.0	22.0
Sex, no			
Male	39	49	5
Female	65	80	8
Illness Duration, days			
Mean ± SD	NA	31.4 ± 23.5	
Median		30.0	
Haemoglobin level, mg ml^-1^			
Mean ± SD	14.26 ± 1.2	8.8 ± 1.5	9.9 ± 1.4
Median	14.5	9.2	10.3
WBC, x10^3^ cells mm^-3^			
Mean ± SD	8941 ± 926	3338 ± 1497	7961 ± 2199
Median	9020	3300	8800
Splenic enlargement, cm			
Mean ± SD	NA	6.0 ± 2.7	0
Median		7.0	

ND, Assay not done

ECs were recruited from people accompanying VL patients to the clinic.

Venous blood was collected from the patients and controls into heparinized tubes. Plasma was separated by centrifugation at 770 g for 10 minutes and stored at -80°C till further use. The plasma was replaced by PBS and PBMC were isolated by density gradient separation using Lymphoprep (STEMCELL Technologies).

Details of all reagents used in this study are described in S1 Table.

### Gene expression for *IL7RA* and *IL7*, mRNA

CD4^+^ cells were enriched from freshly isolated PBMCs by positive selection using anti-human CD4 magnetic Microbeads (Miltenyi Biotec) and MS columns according to the manufacturer’s protocol (Miltenyi Biotec). The enriched CD4^+^ cells were 99% CD4^+^ T cell as analyzed by FACS. After washing the cell pellets, they were stored in RLT buffer at -80°C. Total RNA was isolated from both PBMCs and CD4^+^ cells using the Qiagen RNeasy mini kit following the manufacturer’s instructions. A high-capacity cDNA Reverse Transcription Kit (ThermoFisher) was used to reverse transcribe 1000 ng of RNA according to the manufacturer’s instructions. TaqMan-based gene expression assays were performed for *IL7RA*, *IL7* mRNA targets and 18s ribosomal RNA (rRNA) using 7500 real-time PCR. For each donor, the mean cycle threshold value from duplicated qPCR tests was used to calculate the relative quantification (2^-ΔCt^) as follows:

$$\Delta \text{Ct}=\text{Ct}\;\left(target\;gene\right)-\text{Ct}\;\left(18\text{S}\;\text{rRNA}\right)$$

$$\Delta \Delta \text{Ct}=\Delta \text{Ct}\;\left(\text{Sample}\right)–\Delta \text{Ct}\;\left({\text{EC}}_{\text{mean}}\right)$$

$$\text{Expression}\;\text{ratio}=2{-}^{\Delta \Delta \text{Ct}}$$

As indicated above, the 18S rRNA expression was for internal normalization of each sample. The mean ΔCt of all EC samples (EC_mean_) was used to calculate the fold change between the individual sample and the EC_mean,_ making the spread of samples within the VL and EC groups visible. For each amplification, 25 μg of cDNA was used, each amplification tube containing a mixture of 5 μl of cDNA (5 ng/μl), 1μl of primer/probe, 4 μl of MilliQ, and 10 μl of TaqMan master mix (Applied Biosystems, Foster City, CA, USA).

### Measurement of soluble IL-7 and IL-7Rα in plasma

Plasma samples were thawed at the time of ELISA analysis and diluted two-fold with Assay diluent A (provided in the kit) and concentrations of IL-7 and sIL-7Rα were measured in duplicate using the IL-7 Human ELISA kit (invitrogen) and the Human CD127 ELISA kit (abcam), respectively, following the manufacturer’s protocol. The standard curves were generated using recombinant protein provided by the manufacturer and a 4-parametric logistic regression in SoftMax Pro software (version 3.1.2) to calculate the concentrations of IL-7 and sIL-7Rα.

### Phenotypic expression of IL-7Rα by PBMC staining

For analysis of surface expression, 5 x 10^5^ PBMCs from VL and ECs were used. Briefly, PBMCs were washed with staining buffer (PBS, 5% heat inactivated fetal calf serum) and stained with viability dye Zombie aqua at room temperature for 20 minutes. After washing, surface staining was performed with fluorochrome labelled antibodies against CD3ε, CD4, CD127, CD25, CD45RA, CD185 (CXCR5), CD194 (CCR4), CD196 (CCR6), CD197 (CCR7), CD183 (CXCR3), CD38, and CD132, for 30 minutes at 4°C in the dark. Following washing, the cells were re-suspended in staining buffer, and acquired on a flow cytometer (BD LSRFortessa) using FACS Diva software (version 8.0.2). Flow Jo version 10 software (Tree Star, BD) was used to analyze the FACS data. CD4^+^ T cell subsets were defined as Treg (CD25^+^, CD127^-^), Tem (CD45RA^+/-^, CCR7^-^), Th (Tem—CXCR5), Th17_Th22 (CCR6^+^, CCR4^+^), Th1_Th2 (Th—CCR6, CCR4^+/-^), Th1 (Th1_Th2—CCR4, CXCR3^+^), Th2 (Th1_Th2—CXCR3 CCR4^+^), Th9 (Th—CCR4, CCR6^+^) [23–26]. CD4^+^ T cell subsets were defined as previously reported by us and others (References [18,23–26]), and the gating strategy employed was as we previously described (references [18,25]). Details of all antibodies used in this study are described in S2 Table.

### Signaling potential of IL-7 and IL-7Rα

To assess STAT5 phosphorylation (pSTAT5), as indicative of IL-7Rα signaling capacity in CD4^+^ T cell subsets, freshly isolated heparinized whole blood (200μl) was first surface stained for 5 minutes at room temperature. Thereafter, the samples were stimulated with rhIL-7 (200 ng/ml) for 5 minutes or left unstimulated at 37°C, 5% CO_2_. 200 ng/ml of rhIL-7 was sufficient to induce phosphorylation of STAT5 in maximum CD4^+^ T cells [27]. Phosphorylation of STAT5 was detected using BD Phosflow, according to manufacturer’s instructions. Briefly, the cells were fixed directly after completion with Lyse/Fix Buffer for 7 min at 37°C in a water bath. After washing, the cells were gently vortexed to loosen and permeabilized by using chilled BD Phosflow Perm Buffer III for 30 minutes on ice. The cells were washed twice and stained for intracellular pSTAT5 for 60 minutes at room temperature in the dark, with gentle vortexing every 15 minutes. After washing, the cells were re-suspended in staining buffer acquired on flow cytometer (BD LSRFortessa) using FACS Diva software version 8.0.2.

### Statistical analysis

Statistical analysis was performed using Excel (Microsoft) and GraphPad Prism 8.01 software (Graph Pad Software, La Jolla. CA, USA). Analysis of cellular assays and qPCR was performed using nonparametric Kruskal-Wallis test for multiple groups and with a post test to see between which groups differences exist and Mann-Whitney U-test for comparison between two groups. Wilcoxon signed-rank test was used to compare matched sample pairs. SPICE analysis was performed using SPICE version 5.3 (M. Roeder, Vaccine Research Centre, National Institutes of Allergy and Infectious Diseases, National Institutes of Health, USA; http://exon.niaid.nih.gov) [28]. The data are presented as mean ± SEMs. P-values less than 0.05 were considered statistically significant. Outliers were defined by the ROUT method, alpha 0.05 and removed from analysis.

## Results

### Decrease in soluble sIL-7α and increase in IL-7 plasma protein levels in VL patient

**Fig 1:**
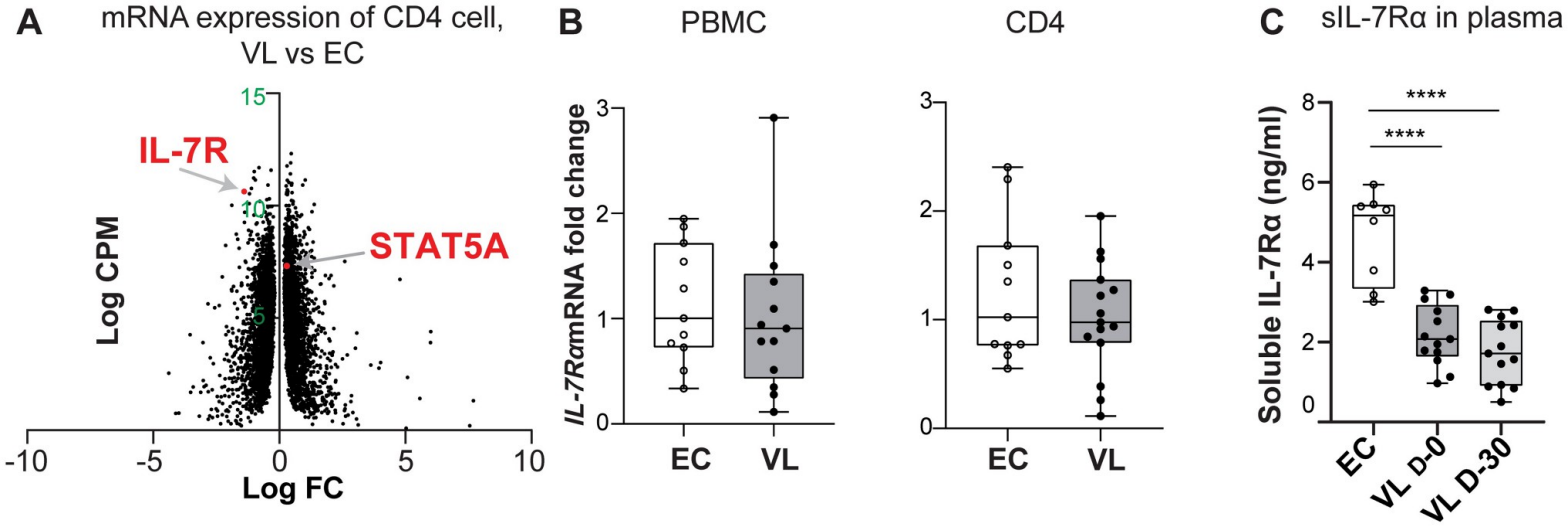
*IL7RA* expression in VL. A. Volcano plot of immune-related genes in peripheral blood CD4^+^ T cell data extracted from a previously published dataset [17]. Analysis of differentially expressed immune-related genes in peripheral blood CD4^+^ T cells between visceral leishmaniasis (VL, n = 12) patients prior to treatment (D0) and endemic controls (EC, n = 12) shows downregulation of *IL7RA*. B. Relative expression of *IL7RA* determined by RT-qPCR in PBMC and CD4^+^ T cells, as indicated, each dot represents one sample PBMC (EC n = 11, VL n = 13), CD4 (EC n = 11, VL n = 15). C. Soluble IL-7Rα plasma levels in EC (n = 8) and VL before (D-0, n = 13) and 30 days (D-30 n = 13) after initiation of drug treatment. Statistical significance was determined by Kruskal-Wallis with multiple comparison follow-up test for Fig 1C and are indicated as *p<0.05; **p<0.01; ***p<0.001; ****p<0.0001.

Aberrant expression of IL-7 and soluble IL-7Rα in plasma is indicative of pathological T cell immunity in chronic viral, inflammatory, and autoimmune diseases. Using data from transcriptional profiling [17] and NanoString mRNA expression analysis [18], we observed down-regulation of *IL7RA* mRNA in CD4^+^ T cells from patients with visceral leishmaniasis (VL) compared to endemic healthy individuals (ECs) (Fig 1A, extracted from [17] and as previously reported [18]). Lower *IL7RA* were also previously reported in VL CD4^+^ T cell pretreatment as compared to post treatment [18]. To confirm this finding, we analyzed the mRNA expression of *IL7RA* in CD4^+^ cells and PBMCs from VL patients using real-time qPCR. Our results did not show any significant difference in expression of *IL7RA* in PBMCs or CD4^+^ cells between VL patients and ECs (Fig 1B). However, in line with the transcriptional profiling data, when, we measured the levels of sIL-7Rα (CD127) in plasma we found that patients infected with *L*. *donovani*, both active infection (D-0) and 30 days post treatment (D-30) had significantly lower levels of sIL-7Rα compared to ECs (p<0.0001) (Fig 1C). Combined, the data indicate an aberrant expression of the IL-7 receptor in VL patients.

**Fig 2:**
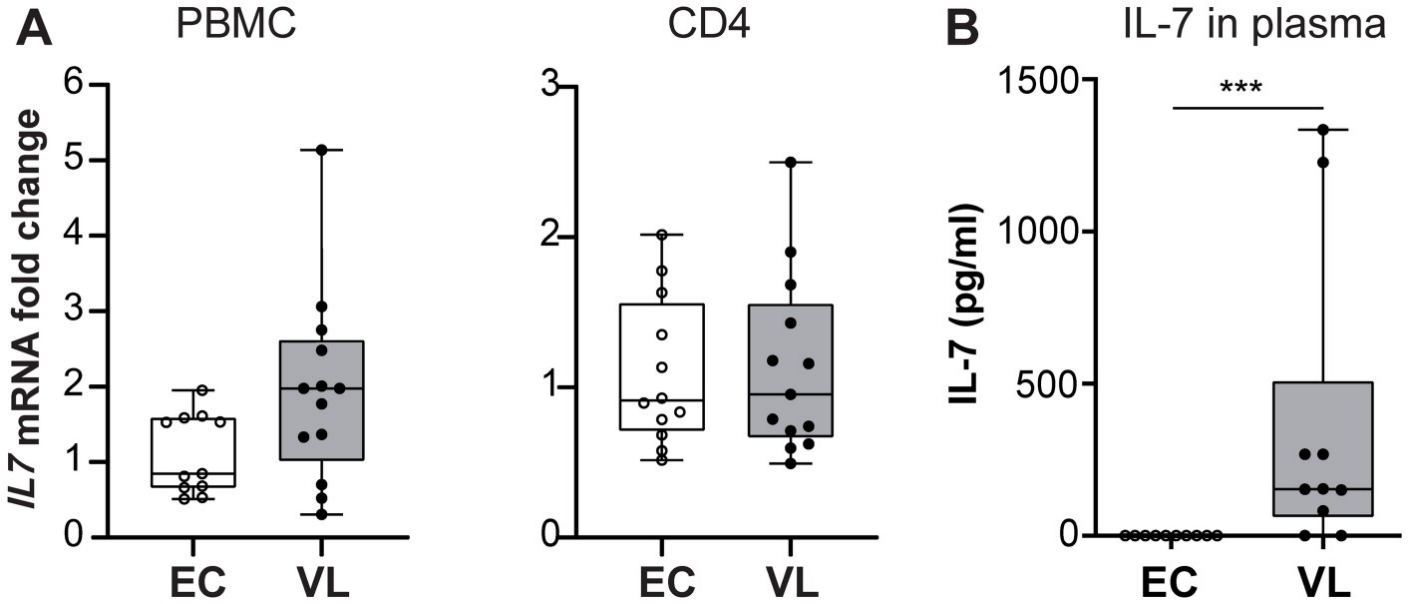
IL-7 expression and secretion following *Leishmania donovani* infection. A. Relative expression of *IL7RA* determined by RT-qPCR in PBMC and CD4^+^ T cells, as indicated, each dot represents one sample, PBMC (EC n = 11, VL n = 13), CD4 (EC n = 12, VL n = 13). Median range is depicted. B. IL-7 levels in the plasma of VL patients (n = 10), ECs (n = 10), as determined by ELISA. Statistical significance was determined by Mann-Whitney U-test for Fig 2A, and Kruskal-Wallis with multiple comparison follow-up test for Fig 2B and are indicated as *p<0.05; **p<0.01; ***p<0.001; ****p<0.0001.

PBMC and CD4^+^ cells are not a major source of IL-7, and analysis of *IL7* mRNA expression did not show any differences between *IL7* mRNA between VL and EC cells (Fig 2A). Surprisingly, analysis of soluble IL-7 in plasma demonstrated that the IL-7 levels were significantly higher in active, VL compared to EC (p<0.001) (Fig 2B). Following treatment of VL, we observed a decrease in the level of soluble IL-7 in plasma (VL D0 (n = 14), 40.13 pg/ml ±19.79 SEM, VL D30 (n = 14) 19.8pg/ml ±12.85 SEM, with P<0.05).

### The IL7 receptor is upregulated on VL CD4^+^ T cells compared to EC

**Fig 3:**
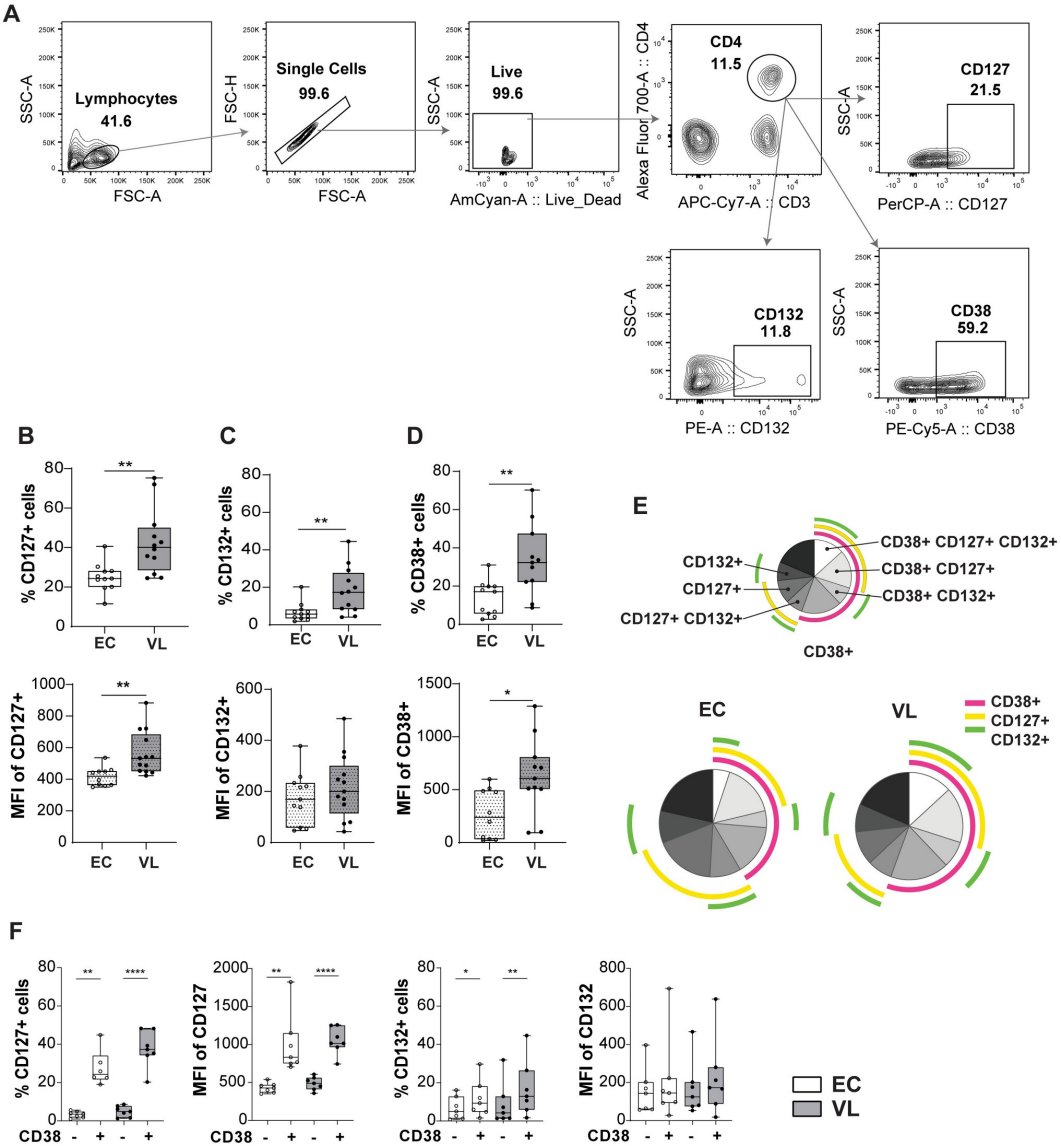
Surface protein expression of CD127 and CD132 on CD4^+^ T cells from endemic controls (ECs) and visceral leishmaniasis (VL) patients. A. Gating strategy for CD4^+^ T cell flow cytometry analysis. B. Percentage (top) and mean fluorescent intensity (MFI) (bottom) of CD127 on CD4^+^ T cells. C. Percentage (top) and MFI (bottom) of CD132 on CD4^+^ T cells. D. Percentage (top) and MFI (bottom) of CD38 on CD4^+^ T cells. Statistical significance between ECs (n = 11) and VL patients (n = 12) was determined by Mann-Whitney U-test in Fig 3B-D, and 3F are indicated as *p<0.05; **p<0.01. E. Boolean-Gating (FlowJo), was used to define complex cell sub-population, and Simplified presentation of incredibly complex evaluations (SPICE) polyfunctionality analysis. SPICE was used to establish overlap in expression of CD38, CD127 and CD132 on CD4^+^ T cells. Pie charts represent the entire CD4^+^ T cell population expressing either CD38, CD127, CD132 or none of them. Data was generated from EC (n = 7) and VL patients (n = 7). F. Percentage and MFI of CD127, and CD132, expression on CD38+/- CD4 T^+^ cells of VL (n = 7) and EC (n = 7).

Next, we conducted an analysis of the surface expression of the IL-7 receptors (CD127 and CD132) on CD4^+^ T cell subsets (as defined in Fig 3A), and observed an upregulation of CD127 and CD132 on VL CD4^+^ T cells (Fig 3B and 3C). Additionally, we detected an increase in activated CD4^+^ T cells in VL patients based on the expression of CD38 (Fig 3D). The activated CD38^+^ CD4^+^ T cells from VL patients expressed higher levels of IL-7Rα, as demonstrated by the higher MFI of CD127 compared to EC and by SPICE analysis (Fig 3D, 3E and 3F) and bar graph (S1 Fig).

**Fig 4:**
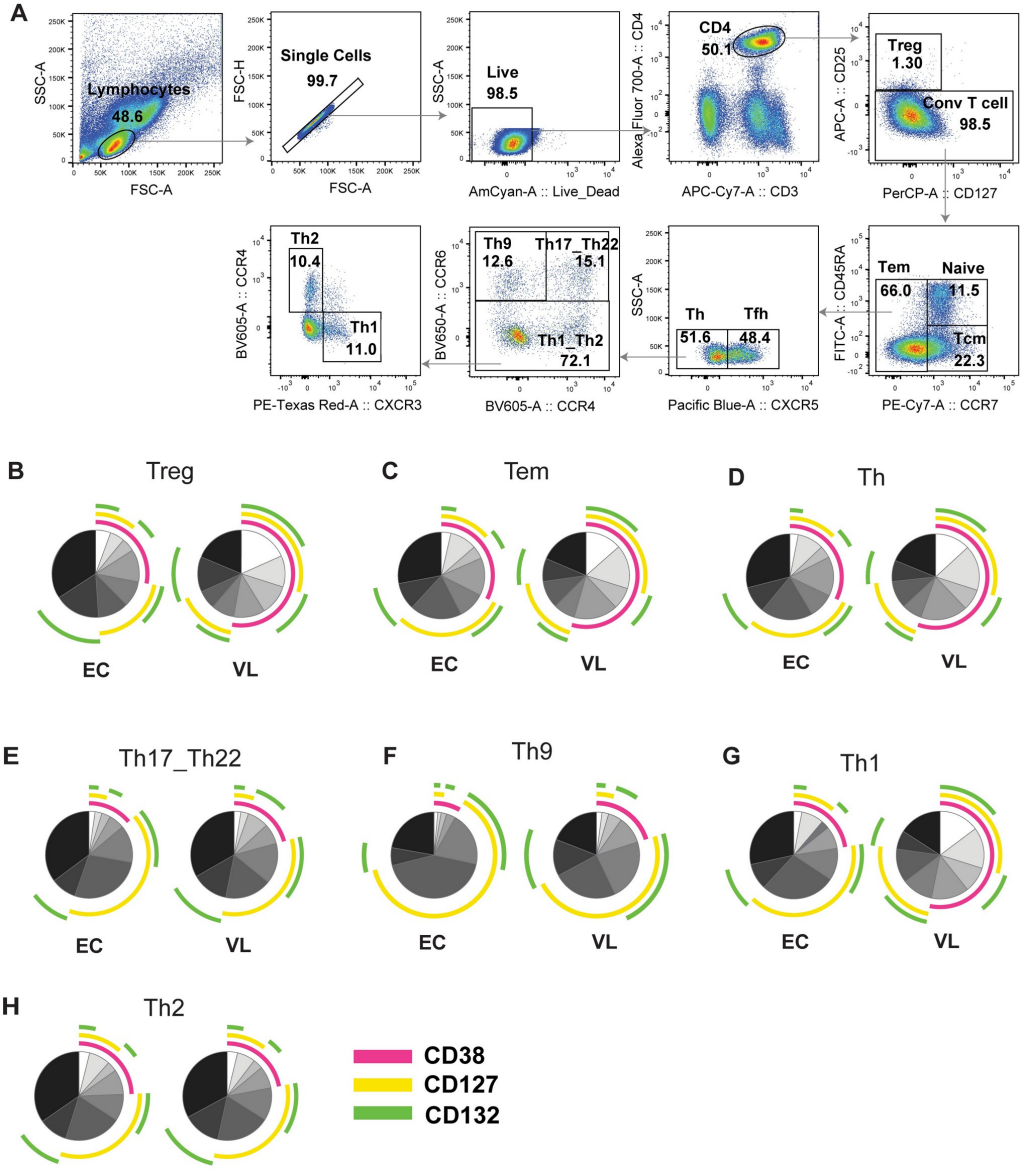
Surface protein expression of CD127 and CD132 on CD4^+^ T cell subsets from endemic controls (ECs) and visceral leishmaniasis (VL) patients. A. Gating strategy for CD4^+^ T cell subsets. B-H. Boolean-Gating and Simplified presentation of incredibly complex evaluations (SPICE) polyfunctionality analysis. SPICE was used to establish overlap in expression of CD38, CD127 and CD132 on CD4^+^ T cell subsets in endemic controls (ECs) and visceral leishmaniasis (VL) patients; EC (n = 7), VL (n = 7). B. Treg cells C. Tem D. Th E. Th17_Th22 F. Th9 G. Th1 H. Th2.

A detailed analysis of IL-7Rα expression on VL patient (D0) CD4^+^ T cells subsets (Fig 4B–4H), (S2 Fig) showed that most Th subsets from VL patients express more, while Th2 and Th17 had similar levels of CD38, CD127 and CD132 compared to ECs.

### VL patient CD4^+^ T cells respond to IL-7 stimulation

**Fig 5:**
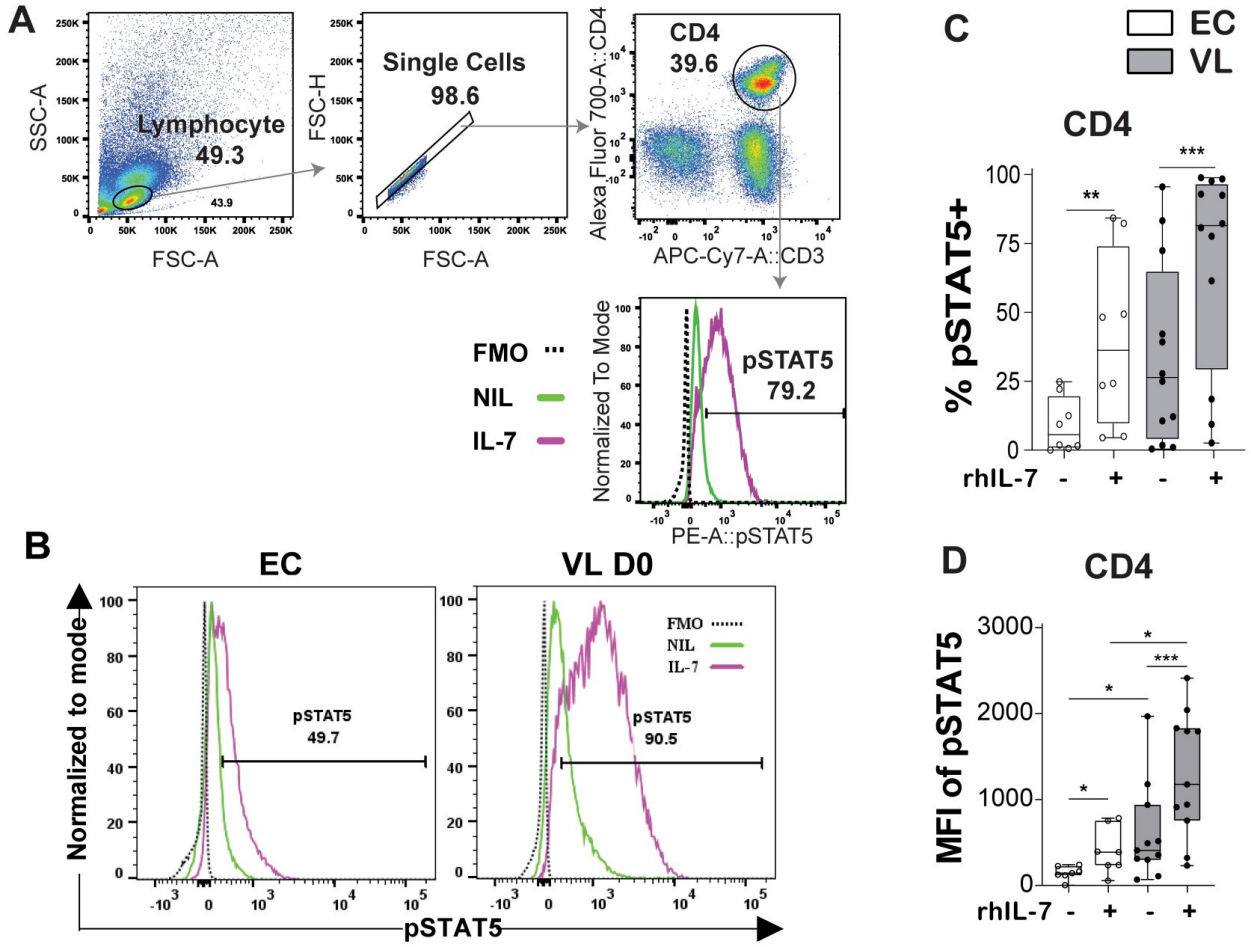
IL-7-mediated activation of intracellular pSTAT5. A. Gating strategy for CD4^+^ T cells. B. Representative histograms of pSTAT5 in endemic controls (ECs) and visceral leishmaniasis (VL) patients following rhIL-7 treatment. C. Frequency of CD4^+^ T cells expressing pSTAT5 and D. Mean fluorescence intensity (MFI) of pSTAT5 in CD4^+^ T cell at baseline without (-) and after rhIL-7 stimulation (+) in EC (n = 8) and VL patients (n = 12). Statistical significance was determined by the Wilcoxon matched-pairs signed rank test between control and rhIL-7 stimulation in figure C-D, or Mann-Whitney U-test to compare EC (n = 8) and VL patients (n = 12) in Fig D. Statistically significant differences are indicated as *P < 0.05; **P < 0.01; ***P < 0.001; ****P < 0.0001.

To test if IL-7 signaling was affected in VL patients we stimulated whole blood with rhIL-7 and measured phosphorylation of STAT5 (pSTAT5), as indicative of IL-7 signaling capacity (Fig 5). Increase in pSTAT5 was most evident in the CD4^+^ T cells, relative to other lymphocyte subsets (S3 Fig). Upon rhIL-7 stimulation pSTAT5 was more noticeable in VL patient as compared to EC CD4^+^ T cells, seen both as frequency of cells positive for pSTAT5 (Fig 5B and 5C) and MFI of pSTAT5 (Fig 5D). Baseline levels (unstimulated cells) of pSTAT5 were higher in VL patient CD4^+^ T cell compared to ECs (Fig 5D), in line with reported higher *STAT5* mRNA levels in VL compared to EC CD4^+^ cells in our previous Nano-String mRNA expression analysis [18].

**Fig 6:**
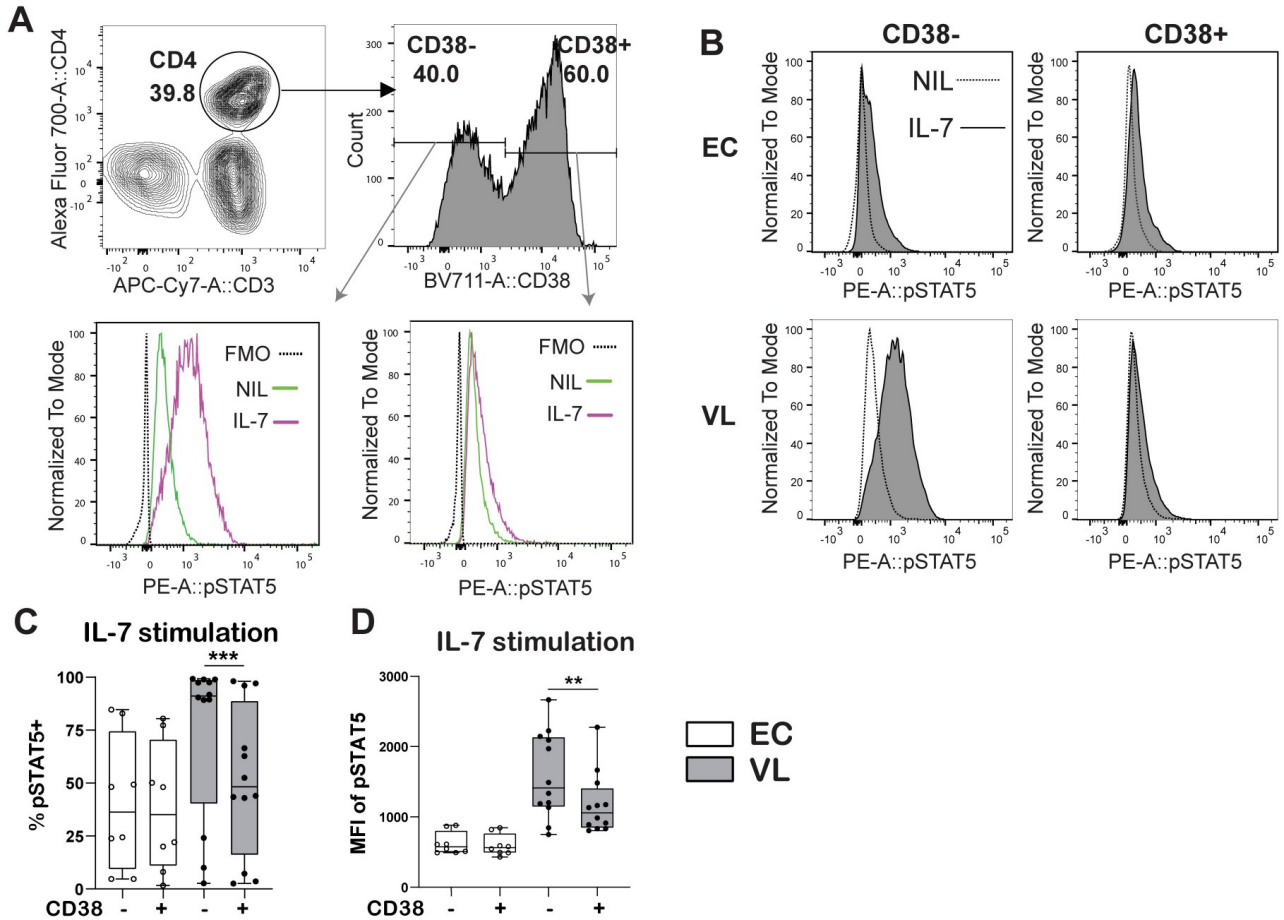
pSTAT5 by CD38^+^ and CD38^-^ CD4^+^ T cells. A-B. Gating Strategy for CD38^+^ and CD38^-^ CD4^+^ T cells. C. Frequency of pSTAT5 expressing CD38^+^ and CD38^-^ CD4^+^ T cells from endemic controls (EC) and visceral leishmaniasis (VL) patients after rhIL-7 stimulation. D. pSTAT5 mean fluorescence intensity (MFI) in CD38^+^ and CD38^-^ CD4^+^ T cells upon rhIL-7 stimulation. Data was generated from EC (n = 8) and VL (n = 12) donor samples. Statistical differences were determined using comparison between two groups with a Wilcoxon matched-pairs signed rank test between control and rIL-7 stimulation and significant differences are indicated as *P < 0.05; **P < 0.01; ***P < 0.001; ****P < 0.0001.

In VL CD4^+^ T cells, pSTAT5 frequency and MFI was increased in all the CD4^+^ Th cell subsets defined upon rhIL-7 stimulation (S4A and S4B Fig). Stimulation with rhIL-7 increased pSTAT5 in CD4^+^ T cell subsets from ECs, but never reached the levels observed in VL patient CD4^+^ T cells (S4A and S4B Fig). To test if IL-7 signaling was linked to activation of T cells, we next examined the IL-7 signaling potential in activated and non-activated CD4^+^ T cells using CD38 as a marker of activation (Fig 6A). In VL patient CD4^+^ T cells pSTAT5 staining was notably stronger in non-activated CD38^-^ compared to activated CD38^+^ CD4^+^ T cells (Fig 6B and 6C), while no differences in pSTAT5 were seen between CD38^-^ and CD38^+^ CD4^+^ T cells in ECs (Fig 6C and 6D).

## Discussion

Lymphopenia and an inability to mount adequate T cell responses contribute to immunosuppression and disease progression in VL patients. Deficiencies in IL-7 signaling have been linked to other chronic diseases such as HIV, and IL-7 therapy has been suggested to improve T cell survival in these patients [29]. Similar to observation made in HIV patients, previous studies by Chauhan *et al*. [18] and Kumar *et al*. [17] found downregulation of *IL7RA* (CD127) in T cells from VL patients compared to ECs [18]. We could not confirm the downregulation in the set of samples included in our analysis here, as no difference in mRNA expression of *IL7RA* between VL patient CD4^+^ T cells and PBMCs, relative to the same cell populations in ECs was observed. The lack of correlation between transcriptional data and RT-qPCR data, was unexpected but may be explained by the use of different individuals in the different assays combined with that the differences in RNA seq and Nanostring results seen between groups were not the most pronounced (logFC -1.39 and -0.719 respectively). However, our analysis of plasma showed less soluble IL-7Rα in the plasma of VL patients both before and 30 days post-drug treatment, compared to ECs, suggesting that the IL-7 pathway could be impaired during *L*. *donovani* infection. The precise biological role of soluble IL-7R (sIL-7Rα) remains unclear, but like the membrane-bound IL7Rα, the sIL-7Rα binds to IL-7 with comparable affinity and is suggested to inhibit IL-7 signaling [30,31]. The reduction in sIL-7Rα was accompanied by increased levels of IL-7 in plasma from VL patients (D0) compared to EC and VL post treatment (D30). Increased plasma level of IL-7 is a sign of lymphopenia [30,31], something frequently observed in VL patients [32]

Higher IL-7 and less sIL-7Rα have also been seen in TB patients [27]. In these patients, the cell surface expression of IL-7Rα and the signaling capacity was reduced in T cells. Transcriptional downregulation of *IL7RA* in T cells that have received IL-7 signaling is a feedback mechanism to prevent competition with T cells that have not yet received the signal [33]. While we found no significant reduction *IL7RA* mRNA in VL by qPCR, previous studies reporting on transcriptional profiling of cells from VL patients found, in line with the observation made in TB patients, reduced *IL7RA* mRNA levels in CD4^+^ T cells from VL patients compared to ECs [17,18]. Interestingly, the surface expression of CD127 and CD132, was found to be increased in VL patients (D0) compared to ECs, this in contrast to TB patients, where IL-7Rα surface expression also was reduced. This finding led us to further investigate IL-7Rα surface expression and the signaling capacity of the receptor on CD4^+^ T cell subsets. Most CD4^+^ T cell subsets from VL patients had more CD127 and CD132 on their surface as compared to ECs. CD38, which is upregulated by inflammatory mediators [34], was used as an activation marker on CD4^+^ T cells (9, 20–22, 29, 31–40). More CD4^+^ T cells expressed CD38 in active VL compared to ECs. These activated (CD38^+^) cells expressed more IL-7Rα as compared to non-activated (CD38^-^) CD4^+^ T cells from VL patients.

Impaired IL-7 signaling via the IL-7Rα, as measured by pSTAT5 levels in T cells has been observed in subjects with HIV infection and TB [35–37]. In contrast, pSTAT5 levels were higher in VL patient CD4^+^ T cells at baseline and after stimulation with rhIL-7, compared to EC CD4^+^ T cells. This finding was surprising since VL is characterized by lymphopenia and dysregulated CD4^+^ T cells, but is in accord with elevated IL-7Rα on the cell surface of VL patient CD4^+^ T cells, and previously reported upregulation of *STAT5* mRNA in VL patient CD4^+^ T cells [18]. The increased IL-7 signaling may be a response to the lymphopenia to support the survival of existing T cells in VL. While we show that additional rhIL-7 stimulation increased (already heightened) pSTAT5 in VL patient CD4^+^ T cells compared to EC, it is uncertain if manipulation of the IL-7 signaling pathway would improve cell survival in VL patients. When comparing pSTAT5 in CD38^+^ compared to CD38^-^ CD4^+^ T cells, less pSTAT5 was seen in the CD38^+^ CD4^+^ T cell subset from VL patients, where the pSTAT5 levels were similar in the two subsets in ECs, suggesting that there is a feedback mechanism to downregulate IL-7 signaling upon activation.

While not conclusive, our attempts to improve cell survival in 72-hour antigen stimulation assays by addition of rhIL-7 were ineffective on both EC (n = 5) and VL (n = 5) cells, measured as frequency of 7AAD, Annexin V positive lymphocytes, moreover the addition of rhIL-7 did not alter the levels of IFNγ in culture supernatants of antigen or superantigen stimulated cells. On the basis of these preliminary findings, we did not pursue these investigations further. However, further investigation into feedback mechanisms regulating IL-7 signaling could be of relevance to understand cell survival and death in lymphopenic conditions. Interestingly, it has been shown in mice that prolonged exposure to high IL-7 levels leads to IFN-γ triggered apoptosis in CD8 T cells, with cells having low affinity T cell receptor (TCR) engagement being particularly affected [38]. Furthermore, Rehti *et al*. have shown that high levels of IL-7 can prime both human T cells and B cells for Fas-mediated apoptosis in [39,40] With elevated expression and secretion of Fas/FasL [41] and IFN-γ [42] being a reported features in VL patients, the high IL-7 levels and the elevated IL-7 signaling assumed to promote cell survival and proliferation could potentially simultaneously prime the T cell for to Fas/FasL induced death. Thus, the lower pSTAT5 seen in VL CD38^+^ compared to CD38^-^ CD4^+^ T cell could potentially be beneficial for the survival of effector T cells in VL.

In conclusion, defects in IL-7Rα expression or IL-7 signaling were not evident in CD4^+^ T cells from VL patients. Instead, VL patient CD4^+^ T cells appeared to maintain an elevated expression of IL-7Rα and pSTAT5, as compared to ECs. Our data does not support impaired IL-7 signaling as an explanation for loss of CD4^+^ T cells during VL. We speculate that the IL-7/IL-7Rα pathway may allow cells to survive longer but render them weakened and susceptible to apoptosis when actively engaged in the immune response.

## Supporting information

S1 Fig Supporting to Fig 3E. Frequency of CD4^+^ T cell expressing CD38, CD127 and/or CD132 as indicated on the Y axis.
(TIF)

S2 Fig Supporting to Fig 4B–4H. Frequency of the gated CD4^+^T cells subsets expressing CD38, CD127 and/or CD132 as indicated on the Y axis.
(TIF)

S3 Fig Gating of lymphocyte populations and representative pSTAT5 in CD4^+^ T cells and other lymphocytes upon rhIL-7 stimulation. pSTAT5 in CD3^+^ CD4^+^ T cells, CD3^+^ CD4^-^ T cells and CD3^-^ CD4^-^ cells in EC and VL.
(TIF)

S4 Fig pSTAT5 expression by CD4^+^ T cell subsets from endemic controls (EC) and visceral leishmaniasis (VL) patients. A. Merged CD4^+^ T cell samples were used to create t-distributed stochastic neighbor embedding (tSNE) plots showing CD38, STAT5 and pSTAT5 expression by CD4^+^ T cells from ECs and VL patients upon rhIL-7 stimulation. Each point represents one single cell and cells in the same cluster represents high similarity in phenotypic expression. FACS data, showing B. Frequency and C. Mean Fluorescence Intensity (MFI) of pSTAT5 in CD4^+^ T cell subsets identified as shown in Fig 4A. The heat map was rendered using the Morpheus tool, and the grid shows quantitative signaling upon rhIL-7 treatment in activated (CD38^+^) and non-activated (CD38^-^) CD4^+^ T cell subsets (columns) from ECs and VL patients (rows).
(TIF)

S1 Table Reagent List. (DOCX)

S2 Table FACS Antibody. (DOCX)
