# Supplementary figures and images for: Altered IL-7 signaling in CD4+ T cells from patients with visceral leishmaniasis
Source: PLoS Negl Trop Dis. 2024 Feb 26;18(2):e0011960. doi: 10.1371/journal.pntd.0011960 (PMC10919868; doi:10.1371/journal.pntd.0011960)

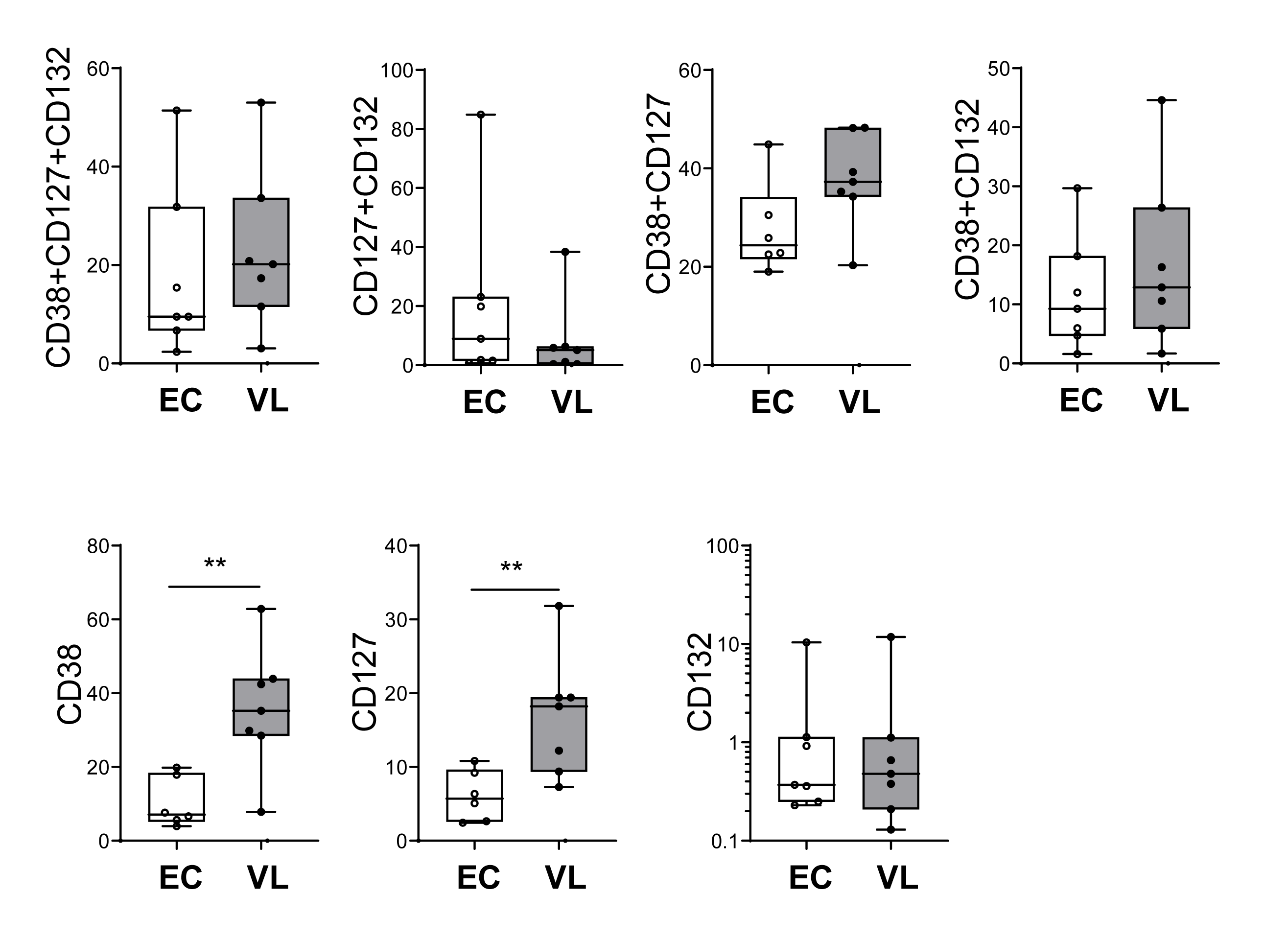

Supplement: S1 Fig — Frequency of CD4+ T cell expressing CD38, CD127 and/or CD132 as indicated on the Y axis. (TIF) [file pntd.0011960.s001.tif]

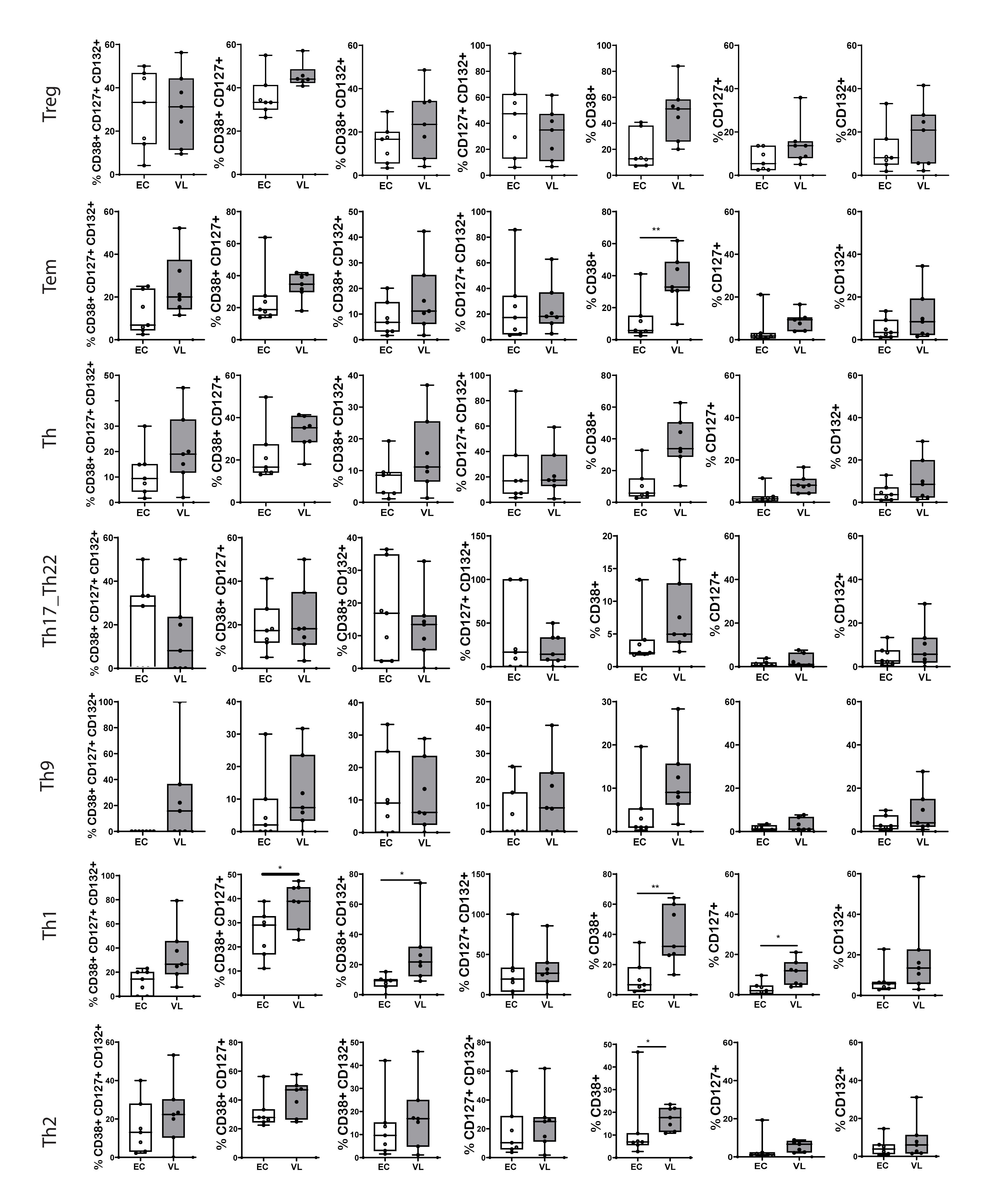

Supplement: S2 Fig — Frequency of the gated CD4+T cells subsets expressing CD38, CD127 and/or CD132 as indicated on the Y axis. (TIF) [file pntd.0011960.s002.tif]

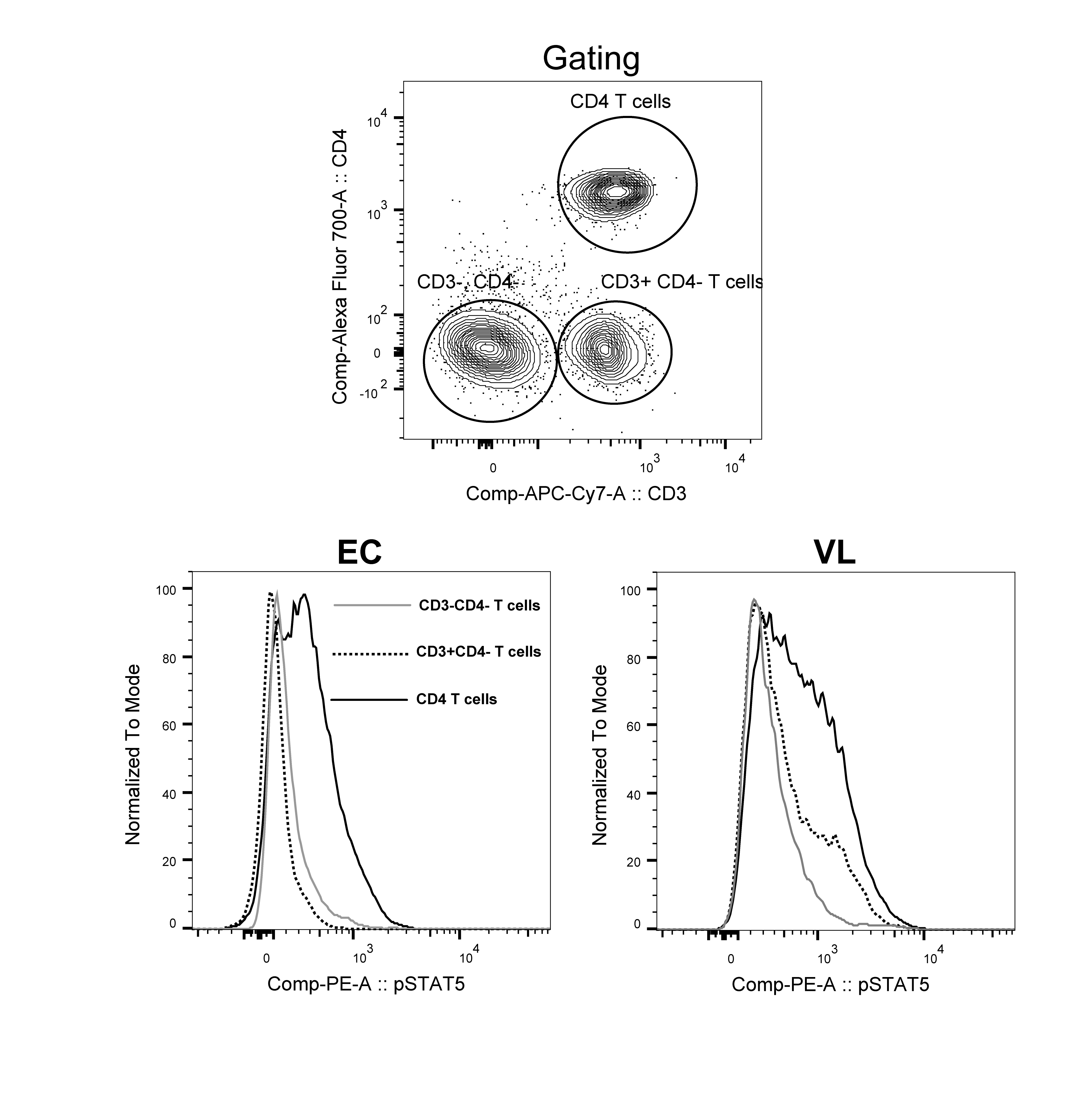

Supplement: S3 Fig — pSTAT5 in CD3+ CD4+ T cells, CD3+ CD4- T cells and CD3- CD4- cells in EC and VL. (TIF) [file pntd.0011960.s003.tif]

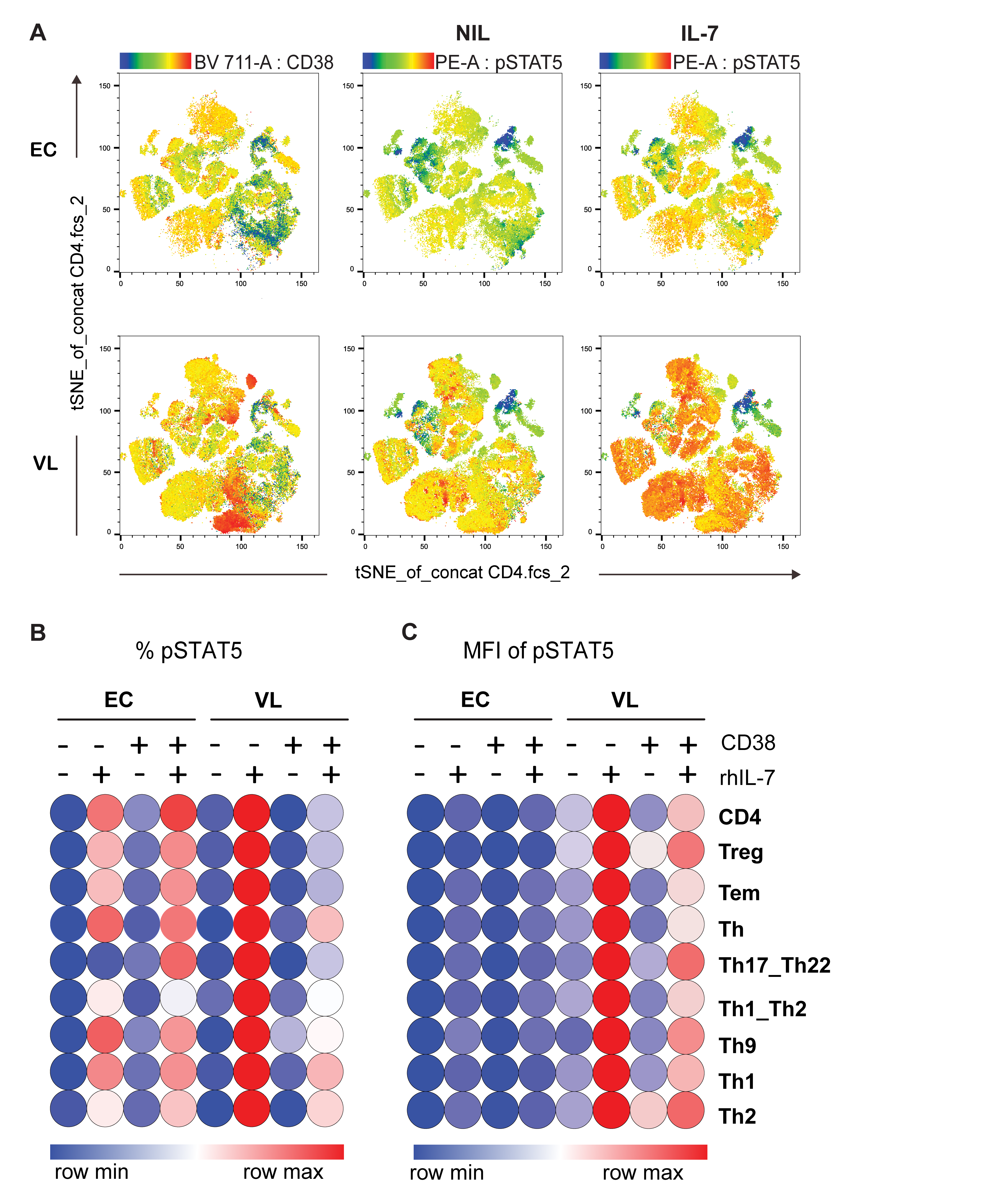

Supplement: S4 Fig — A. Merged CD4+ T cell samples were used to create t-distributed stochastic neighbor embedding (tSNE) plots showing CD38, STAT5 and pSTAT5 expression by CD4+ T cells from ECs and VL patients upon rhIL-7 stimulation. Each point represents one single cell and cells in the same cluster represents high similarity in phenotypic expression. FACS data, showing B. Frequency and C. Mean Fluorescence Intensity (MFI) of pSTAT5 in CD4+ T cell subsets identified as shown in Fig 4A. The heat map was rendered using the Morpheus tool, and the grid shows quantitative signaling upon rhIL-7 treatment in activated (CD38+) and non-activated (CD38-) CD4+ T cell subsets (columns) from ECs and VL patients (rows). (TIF) [file pntd.0011960.s004.tif]
