# Supplementary material for: Altered IL-7 signaling in CD4+ T cells from patients with visceral leishmaniasis
Source: PLoS Negl Trop Dis. 2024 Feb 26;18(2):e0011960. doi: 10.1371/journal.pntd.0011960 (PMC10919868; doi:10.1371/journal.pntd.0011960)
Supplement: S1 Table — (DOCX) [file pntd.0011960.s005.docx]

S1 Table. Reagent list

| Sl no. | **Item** | **Catalogue no** | **Make** |
| --- | --- | --- | --- |
| 1 | PBS, pH 7.4 | 10010023 | ThermoFisher |
| 2 | Lymphoprep | 7861 | STEMCELL, Germany |
| 3 | Recombinant Human IL-7 (carrier-free) | 581906 | Biolegend |
| 4 | CD4 MicroBeads, human | 130-045-101 | Miltenyi Biotec |
| 5 | RNeasy Micro Kit | 74004 | Qiagen |
| 6 | High-Capacity cDNA Reverse Transcription Kit with RNase Inhibitor | 4374966 | ThermoFisher |
| 7 | TaqMan™ Gene Expression Master Mix | 4369510 | ThermoFisher |
| 8 | MS columns | 130-042-201 | Miltenyi Biotec |
| 9 | IL-7 Human ELISA Kit | EHIL7, | Invitrogen |
| 10 | CD127 ELISA Kit | ab213799, | abcam |
| 11 | BD Phosflow (Lyse/Fix buffer 5x) | 558049 | BD Biosciences |
| 12 | BD Phosflow (Perm Buffer III) | 558050 | BD Biosciences |
| 13 | IL-7 Primer | Assay ID Hs00174202_m1 Catalogue:  4331182 | ThermoFisher |
| 14 | IL-7R Primer | Assay ID Hs00902334_m1 Catalogue: 4331182 | ThermoFisher |
| 15 | 18S rRNA Primer | Assay ID Hs99999901_s1, Catalogue: 4331182 | ThermoFisher |
