# Supplementary material for: Altered IL-7 signaling in CD4+ T cells from patients with visceral leishmaniasis
Source: PLoS Negl Trop Dis. 2024 Feb 26;18(2):e0011960. doi: 10.1371/journal.pntd.0011960 (PMC10919868; doi:10.1371/journal.pntd.0011960)
Supplement: S2 Table — (DOCX) [file pntd.0011960.s006.docx]

S2 Table. FACS Antibody

| Sl no. | FACS Antibody | Color | Clone | Make |
| --- | --- | --- | --- | --- |
| 1 | CD3 | APC Cy7 | SK7 | BD Bioscience |
| 2 | CD4 | AF 700 | RPA-T4 | BD Bioscience |
| 3 | CD127 | PerCp | HIL-7R-M21 | BD Bioscience |
| 4 | CD132 | PE | AG184 | BD Bioscience |
| 5 | CD45RA | FITC | HI 100 | BD Bioscience |
| 6 | CD183 | PE Texas Red-A | IC6 | BD Bioscience |
| 7 | CD25 | APC | 2A3 | BD Bioscience |
| 8 | CD185 | Pacific Blue | RF8B2 | BD Bioscience |
| 9 | CD196 | BV650 | 11A9 | BD Bioscience |
| 0 | CD38 | PE Cy5 | HIT2 | BD Bioscience |
| 11 | CD197 | PeCy7 | 3D12 | BD Bioscience |
| 12 | CD194 | BV605 | 291H4 | BioLegend |
| 13 | Zombie Aqua | AmCyan | - | eBiosciences |
| 14 | p STAT5 | PE | 47/Stat5(pY694) (RUO) | BD Bioscience |
